# Insight into OroxylinA-7-*O*-β-d-Glucuronide-Enriched *Oroxylum indicum* Bark Extract in Oral Cancer HSC-3 Cell Apoptotic Mechanism: Role of Mitochondrial Microenvironment

**DOI:** 10.3390/molecules26247430

**Published:** 2021-12-07

**Authors:** Sharmila Kameyanda Poonacha, Madhyastha Harishkumar, Madhyastha Radha, Remya Varadarajan, Suchetha Kumari Nalilu, Shilpa Sharathraj Shetty, Praveen Kumar Shetty, Revanasiddappa Bistuvalli Chandrashekharappa, Mahendra Gowdru Sreenivas, Satheesh Kumar Bhandary Bavabeedu

**Affiliations:** 1 Central Research Laboratory, K.S. Hegde Medical Academy, Nitte (Deemed to Be) University, Mangaluru 575018, India; sharmila@nitte.edu.in (S.K.P.); remyavaradarajan18@gmail.com (R.V.); kumarin@nitte.edu.in (S.K.N.); shilpajshetty@nitte.edu.in (S.S.S.); praveenkumarshetty@nitte.edu.in (P.K.S.); 2 Department of Cardio-Vascular Physiology, Faculty of Medicine, University of Miyazaki, Miyazaki 8891692, Japan; radharao@med.miyazaki-u.ac.jp; 3 Department of Biochemistry, K.S. Hegde Medical Academy, Nitte (Deemed to Be) University, Mangaluru 575018, India; 4 Department of Pharmaceutical Chemistry, NGSM Institute of Pharmaceutical Sciences, Nitte (Deemed to Be) University, Mangaluru 575018, India; revan@nitte.edu.in (R.B.C.); mahimahendrags@gmail.com (M.G.S.); 5 Department of Otorhinolarynology, K.S. Hegde Medical Academy, Nitte (Deemed to Be) University, Mangaluru 575018, India

**Keywords:** *Oroxylum indicum*, HSC-3 cell, oral cancer, mitochondrial stress, mitochondrial membrane potential, apoptosis, ROS

## Abstract

*Oroxylum indicum*, of the Bignoniaceae family, has various ethnomedical uses such as an astringent, anti-inflammatory, anti-bronchitis, anti-helminthic and anti-microbial, including anticancer properties. The druggability of OI stem bark extract was determined by its molecular docking interactions with PARP and Caspase-3, two proteins involved in cell survival and death. Note that 50 µg/mL of *Oroxylum indicum* extract (OIE) showed a significant (*p* < 0.05%) toxicity to HSC-3 cells. MTT aided cell viability and proliferation assay demonstrated that 50 µg/mL of OIE displayed significant (*p* < 0.5%) reduction in cell number at 4 h of incubation time. Cell elongation and spindle formation was noticed when HSC-3 cells were treated with 50 µg/mL of OIE. OIE initiated DNA breakage and apoptosis in HSC-3 cells, as evident from DNA ladder assay and calcein/EB staining. Apoptosis potential of OIE is confirmed by flow cytometer and triple-staining (live cell/apoptosis/necrosis) assay. Caspase-3/7 fluorescence quenching (LANCE) assay demonstrated that 50 µg/mL of OIE significantly enhanced the RFU of caspases-3/7, indicating that the apoptosis potential of OIE is probably through the activation of caspases. Immuno-cytochemistry of HSC-3 cells treated with 50 µg/mL of OIE showed a significant reduction in mitochondrial bodies as well as a reduction in RFU in 60 min of incubation time. Immunoblotting studies clearly showed that treatment of HSC-3 cells with OI extract caused caspase-3 activation and PARP deactivation, resulting in apoptotic cell death. Overall, our data indicate that OIE is an effective apoptotic agent for human squamous carcinoma cells and it could be a future cancer chemotherapeutic target.

## 1. Introduction

A long-established history of alternative medicine from plant origin has continuously been practiced in southeastern countries since ancient times. *Oroxylum indicum*, commonly known as the trumpet tree (L.) Benth ex Kurz., is a medicinal plant that is found in India, Thailand, Vietnam, Malaysia, Indonesia, the Philippines, China, and Japan. It may reach a height of 8–15 m and has a thick bark [1]. This plant has multiple benefits, with dual purpose as a food source mainly from fruits and seeds or as medicine with parts of the plant. The bark is a light greyish color with a soft and spongy texture that contains horny lenticels. The bark, leaves, fruits, and seeds of the plant are said to have a wide range of biological activities and have already been used in complementary medicine to treat human ailments. The plant cumulatively exerts antibacterial, anti-hyperglycemic, pro-neurogenesis, cardioprotective, anti-adipogenesis, anti-inflammatory, anticancer properties [2] with actions like an astringent, carminative, blood purifier, diuretic, and laxative. In our previous study, we found rich content of OroxylinA-7-*O*-β-d-glucuronide from the ethanolic extract of *Oroxylum indicum* (OI) bark, as confirmed by LC-MS [3]. The biological potential of OI stem bark extracts has been continuously evaluated through scientific investigation as antioxidants [4], antimicrobials [5,6], and cytotoxicity in tumour cell lines such as B-16 (murine melanoma), HCT-8 (human colon carcinoma), CEM, and HL-60 (leukemia) [7] and HeLa cells [8]. As far as the anticancer property of OI bark extract is concerned, not much research has been done and, as such, the mechanism of its cytotoxic activity is yet to be established. OroxylinA-7-*O*-β-d-glucuronide is a flavonoid derivative and has a principal effect as radical scavenging properties with potent anticancer aspects. Flavonoid modified drug (FMD) is the recent class of anticancer drugs with a better effect on lung cancer [9]. Very little and sporadic information is available on the biodistribution of Oroxylum uptake and biodistribution in the cancer tissue. In HepG2 cell lines, uptake and distribution tests revealed a time-dependent transport property. Furthermore, after being taken up by hepatic tumor cells, both OroxylinA and OroxylinA 7-*O*-d-glucuronide (OG) were primarily dispersed into nuclei [10]. Furthermore, OG was observed in mitochondria, indicating that OG may have an additional target in hepatic cancer cells.

Mitochondria are vital components of living cells, serving as both an energy source and a source of reactive substrates that are detrimental to cells. The mitochondria of cancer cells change physically and functionally from those of normal cells according to cellular homeostasis [11,12]. Furthermore, tumor cells undergo substantial metabolic reprogramming, making them more vulnerable to mitochondrial disruption than non-immortalized cells [13,14]. Mitochondria are recognized to play a significant part in the complicated apoptotic pathway, causing cell death in a variety of ways such as interrupting electron transport-dependent energy metabolism, releasing or activating apoptotic proteins, and changing the cellular redox potential [15,16,17]. The use of pharmacological drugs that cause or promote mitochondrial membrane permeabilization to rectify cancer-related mitochondrial dysfunctions and (re)activate cell death programs represents appealing cancer therapeutic options [18,19,20]. As a result, more studies are in close attention to mitochondria for developing anticancer therapies based on mitochondrial targeting. To mention a few, these targets include mtDNA, the mitochondrial respiratory chain, permeability transition pore complex (mPTPC)-controlled mitochondrial membrane permeabilization, potassium channels on mitochondria, and numerous mitochondria-associated anti- and pro-apoptotic factors [21,22,23].

Mitochondrial reactive oxygen species (mtROS)-induced oxidative stress can result in fast depolarization of the inner mitochondrial transmembrane potential (MTP) and oxidative phosphorylation (OXPHOS) disruption [24]. High amounts of ROS, such as those produced by many chemotherapy medications, have been demonstrated in investigations to have a lethal effect on cancer cells and cause apoptosis via disruption of the mitochondrial membrane and function [25,26]. Extrinsic (death receptor) and intrinsic (mitochondrial-mediated) mechanisms are used to induce apoptosis [27,28]. The intrinsic route controls the permeability of the mitochondrial outer membrane and generates pores in it, allowing apoptosis-inducing substances like cyt c to leak into the cytoplasm constantly. Cyt c and caspase-9 form an apoptosome, which then activates caspase-3, resulting in apoptosis and death. It is worth emphasizing that mtROS accumulation occurs before MTP (m) damage, nuclear condensation, and the creation of apoptotic bodies [29].

In the buccal cavity microenvironment, squamous cell carcinoma (SCC) is a life-threatening type of skin cancer associated with tobacco use, alcohol abuse, and human papillomavirus (HPV) infections. In the present study, for the first time, we report the in vitro cell pharmacokinetics profiles of OroxylinA-7-*O*-β-d-glucuronide enriched *Oroxylum indicum* (OI) bark extract in squamous cell carcinoma HSC-3 cell line and reveal the significance of mitochondrial associated apoptotic mechanism. Cancer cell acquires resistance against apoptosis. Cancer cells exhibit apoptosis resistance to sustain their uncontrolled proliferation, hence any drug that inhibits apoptosis is desired as a potential cancer chemotherapeutic agent [30]. Low levels of reactive oxygen species (ROS) are known to support cells to survive, while an increase in ROS causes cell cycle arrest and death [31]. ROS-modulating therapies are being proposed as therapeutic strategies to preferentially target cancer cell death [32].

We investigated the molecular interaction of major phytochemicals in OI stem bark extract identified in Liquid Chromatography–Mass Spectroscopy (LC–MS) [3] using molecular docking studies to evaluate their binding affinities with targeted apoptosis protein (caspase-3) and PARP. Our study demonstrated effective cytotoxicity and apoptosis by *O. indicum* stem bark extract in HSC-3 cells, achieved by the induction of mtROS and the disruption of mitochondrial membrane potential.

## 2. Results and Discussion

The focus of this research was to understand what mechanism might be at work in *Oroxylum indicum* bark extract’s anticancer properties.

### 2.1. Molecular Docking

**Figure 1:**
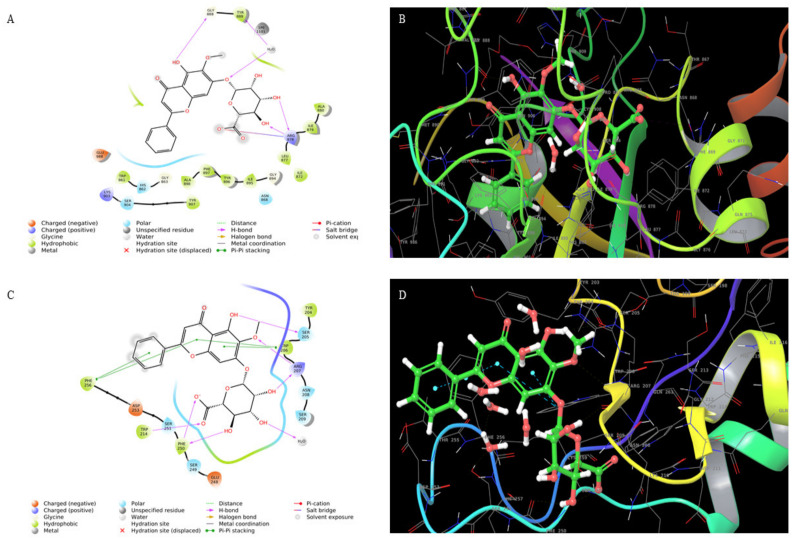
(**A**) Interaction of oroxylin-A-7-*O*-Beta-d-Glucuronide with PARP and (**B**) 3D structure. (**C**) Interaction of oroxylin-A-7-*O*-Beta-d-Glucuronide with caspase-3 and (**D**) 3D structure.

In silico studies carried out using the compound Oroxylin-A-7-*O*-beta-d-glucuronide identified in the *Oroxylum indicum* bark extract docked effectively against PARP (Figure 1A,B) and caspase-3 proteins (Figure 1C,D), suggesting the possible induction of apoptosis in HSC-3 cancer cells. Molecular docking studies have been performed in the groove of the target binding site of PARP (PDB ID: 5DS3) and caspase-3 (PDB ID:2C1E). Oroxylin-A-7-*O*-beta-d-glucuronide has shown the finest binding energies with PARP and caspase-3 with a binding score of −9.689 (Figure 1B,C) and −7.636, respectively.

### 2.2. Cell Viability and Proliferation

**Figure 2:**
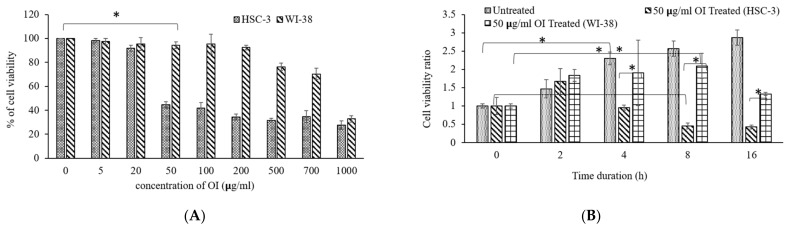
(**A**) Cytotoxicity of *Oroxylum indicum* stem bark extract against HSC-3, human oral squamous carcinoma cell line in a dose-dependent manner; (**B**) Cell viability ratio observed at different incubation periods in hours (0, 2, 4, 8, 16) on exposure to 50 μg/mL of OI extract. * *p* < 0.05 OI treatment v/s control.

The cytotoxic impact of *Oroxylum indicum* bark extract was assessed using the MTT test. Cisplatin (15 ng/mL), a known anticancer drug, was used as a positive control [33]. There was a dose-dependent decrease (Figure 2A) in cell viability on OI treatment for 16 h. The IC_50_ of OI extract against the HSC-3 cell line was found to be 50 μg/mL. The efficacy of OI extract against the HSC-3 cell line at varied concentrations is illustrated (Figure 2A). Since 50 μg/mL OI treatment resulted in considerable lethality to cell, this dose was used in subsequent cell-line-based anticancer studies. Of note, 50 μg/mL of OI extract were incubated for different time intervals of 0, 2, 4, 8, 16 h to analyze the time period at which significant decrease in cell viability ratio is observed. Significant decrease in cell population ratio was observed at 8 h of incubation (Figure 2B). The OI extract’s cytotoxicity was limited to the HSC-3 oral squamous cell carcinoma cell line and not to the control WI-38 cells. We concluded that OI 50 μg/mL dose at 8 h of incubation is an optimum lethal dose to HSC-3 cell than the WI-38 cell, which confirms the bio-model action of OI extract.

### 2.3. Cell Morphology Analysis

**Figure 3:**
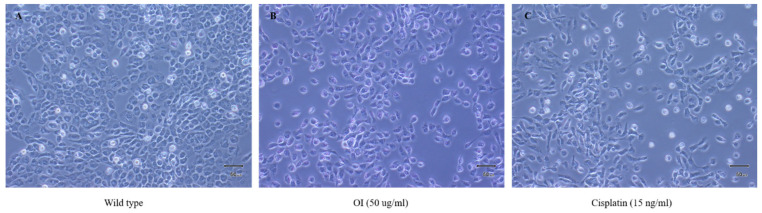
Morphological changes observed in HSC-3, human oral squamous carcinoma cell line; (**A**) wild type which showed epithelial—like charateristics (**B**) OI extract (50 μg/mL): lost epithelial-like appearance and (**C**) Cisplatin (15 ng/mL): lost epithelial-like appearance.

Untreated control HSC-3 cells showed epithelial-like morphological characteristics (Figure 3A). The wild-type cells were uniform in their size and shape and were closely packed. HSC-3 cells treated with OI extract (50 μg/mL) displayed considerable visual changes, as they lost their epithelial-like appearance. The treated cells also lost contact with neighboring cells, reduced in size, and took on a rounded form, and cell size and number reduced (Figure 3B). Cisplatin, a proven anticancer drug, also showed similar changes as OI-treated cells (Figure 3C).

Using a light microscope, the morphological features of the cells were observed and photographed at a magnification of 40×. The control cells in the left panel (Figure 3A) have an epithelial-like shape, which is typical of HSC-3 cells. The cells in the center (Figure 3B) and right panel (Figure 3C) were treated with OI extract (50 µg/mL) and Cisplatin (15 ng/mL) for 8 h, respectively. There were noticeable morphological alterations, such as loss of contact with neighboring cells, significant reduction in size, a decrease in cell number, and a rounded form. This result confirms that cells treated with OI lost contact phenomenon and cell-to-cell communication and probably started to grow in cell coma stage.

### 2.4. OI Induces Apotosis in HSC-3 Cell

**Figure 4:**
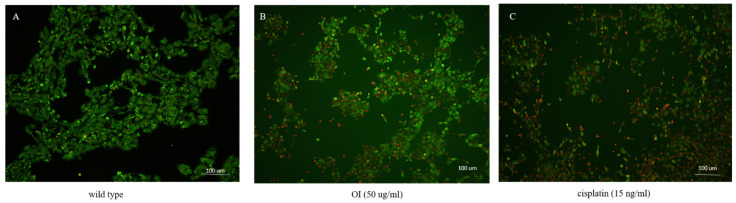
A live/dead cell assay after treatment of HSC-3 cells with the OI extract for 16 h. (**A**) Wild type: most of the cells showed green fluorescence, denoting viable cells stained with calcein-AM. (**B**) OI (50 μg/mL)-treated cells showed cytotoxic activity, represented as more red dead cells stained with Ethidium Bromide. (**C**) Cisplatin (15 ng/mL) treatment also showed more red cells, indicating dead cells due to the cytotoxic activity of cisplatin.

A live/dead cell assay was performed to confirm the cytotoxic activity of *Oroxylum indicum* stem bark extracts against the HSC-3 cell line. Cells were treated with a concentration of 50 μg/mL of OI extract for 8 h. Then, dual staining with fluorescence dyes was performed using calcein-AM and Ethidium bromide. Wild-type cells were green, indicating live cells (Figure 4A). The results showed a significant decrease in viable cells after being treated with the OI extract (Figure 4B), as indicated by red fluorescence. These results suggest that the OI stem bark extract showed potential cytotoxic activity against the studied cell line (HSC-3).

### 2.5. DNA Fragmentation Is a Phenomenon Characterised by OI

**Figure 5:**
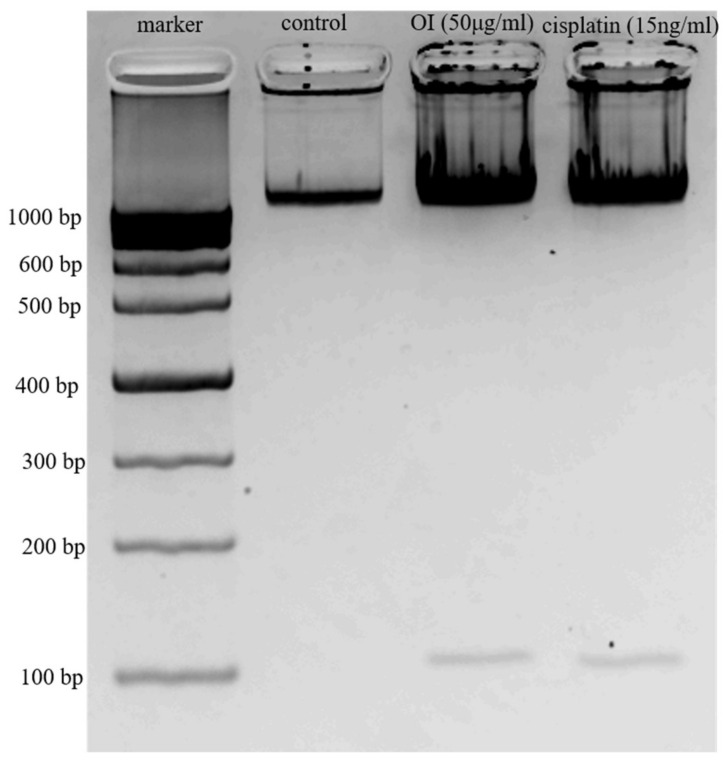
DNA Ladder Assay. Lane 1, 1000 kb marker; Lane 2, Control—DNA from HSC-3 cells; Lane 3, DNA from HSC-3 cells treated with 50 μg/mL of OI extract; Lane 4, DNA from HSC-3 cells treated with 15 ng/mL of Cisplatin.

HSC-3 cells were treated with 50 μg/mL of OI, and cells were resolved in 1.0% agarose gels (Figure 5). DNA ladder pattern of fragmentation was observed. These data suggest that *Oroxylum indicum* is a potent inducer of apoptosis in HSC-3 cells. No bands were observed in the control lane, whereas OI and CP treatment showed two bands indicating DNA breakage phenomena. The upper dark band observed in OI and Cisplatin indicates undigested DNA. The presence of DNA laddering in the OI extract-treated HSC-3 cells compared to the untreated control cells suggests that apoptosis was involved in cell death (Figure 5). Apoptotic cell death is identified by the presence of a specific DNA ladder in cancer cells after anticancer treatment [34,35].

Electrophoretic examination of apoptotic cells reveals particular DNA cleavage as a characteristic ladder pattern resulting in clear DNA fragments of oligonucleosomal size (180–200 bp) [36]. Because ladder production may only be clearly visualized when the level of oligonucleosomal breakage is significant during the late stage of apoptosis, the DNA ladder technique has a reduced sensitivity for apoptosis detection [37]. Hence, more specific apoptosis detection methods including FACS, caspase assays, DAPI staining, Western blotting were conducted to confirm the more specific action of OI.

### 2.6. Apoptosis Assay—(FACS) Flow Cytometry Analysis

**Figure 6:**
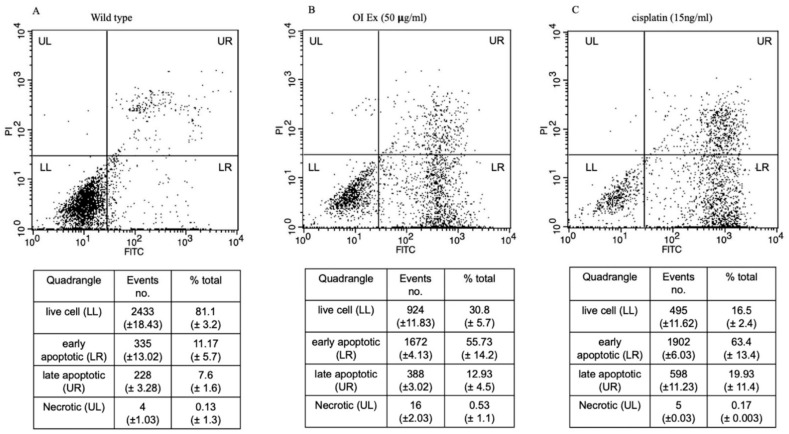
Dot plots of Annexin V-FITC and PI staining to evaluate apoptosis HSC-3 cells following *O. indicum* treatment. HSC-3 cells were treated with 50 μg/mL OI for 8 h, then incubated with annexin V-FITC and PI, and flow cytometry was used to examine the results. (**A**) Control—cells that are negative for both PI and annexin V-FITC are shown in the lower-left quadrant. (**B**) OI extract treatment—annexin positive cells (early apoptotic) in the lower right quadrant, annexin and PI-positive cells in the upper right quadrant (late apoptosis cells). (**C**) Cisplatin (15 ng/mL) treatment—Annexin positive cells in the lower right quadrant, and annexin and PI-positive cells in the upper right quadrant.

The results, shown in Figure 6, reveal that *Oroxylum indicum* tends to induce both early and apoptotic features in HSC-3 cancer cells. The quadraplate scatterplot (Figure 6A–C) represents the percentage of cells showing both early and late apoptotic features for both OI and Cisplatin treatment in HSC-3 cancer cell lines. The apoptosis assay shows different stages of cell death; live cell (LL), early apoptotic (LR), late apoptotic (UR), and necrotic (UL). After 50 μg/mL OI treatment (Figure 6B), the percentage of total live cells was 30.8%, early apoptotic cells were 55.73%, late apoptotic cells were 12.93% and necrotic cells were 0.53%. After 15 ng/mL CP treatment (Figure 6C), the percentage of the total live cells was 16.5%, early apoptotic cells were 63.4%, late apoptotic cells were 19.93% and necrotic cells were 0.17%. When compared to control (Figure 6A), the percentage of early apoptotic cells was significantly higher, indicating apoptosis induction by OI extract.

The Annexin V-FITC/PI apoptosis detection assay, which is widely used to distinguish living cells from both early and late apoptosis [38], was utilized to confirm apoptosis induction. The presence of early and late apoptotic cell populations, as well as no necrotic cells, was observed after OI treatment, indicating that *O. indicum* extract induced apoptotic cell death. Propidium iodide is a fluorescent dye that binds to DNA and cannot penetrate live cells [39]. A rise in propidium iodide fluorescence (red colour) in OI-treated cells correspond to an increase in the number of dead cells. Only cells with broken membranes can stain nucleic acids with propidium iodide [40]. After passing through damaged membranes, propidium iodide can bind to DNA in nonviable cells. As a result, the loss of membrane integrity may be attributed to the *O. indicum* extract, as it was not seen in the control cells. The results gave more insights to determine whether OI extract promotes apoptosis and, if so, how much apoptosis is induced. In actuality, the main mechanism for most anticancer medicines is the stimulation of apoptotic signaling pathways to cause cancer cell death [41]. One of the early events in apoptosis is that cells that are undergoing apoptosis reorient phosphatidylserine from the inner side of the plasma membrane to its outer leaflet. Phosphatidylserine exposure on the cell membrane’s external surface is widely acknowledged as one of the indicators of apoptosis [42]. Cells can bind to annexin V in this environment, and this process can be employed as an apoptosis marker [43]. Using annexin V staining, we observed that, at a concentration of 50 μg/mL, OI plant extract induced apoptosis in HSC-3 cells. Overall, we believe that the anticancer activities of *O. indicum* plant extracts against HSC-3 cells are mediated, at least in part, by cell apoptosis promotion.

The development of methods that selectively restore the apoptotic machinery within tumor cells could be a useful cancer management technique. The primary mode of action of most anticancer medicines is to induce apoptosis in neoplastic cells by inducing apoptotic pathways in altered cells during the carcinogenesis process [44,45,46,47].

The mitochondrial route includes cytochrome c (cyt. c), apoptotic protease activating factor 1, and caspase-9 [48,49]. Apoptosis is primarily mediated by two signaling routes, the mitochondrial and death receptor pathways. The mitochondrial pathway is implicated in the function of a majority of anticancer drugs. Activated caspase-3 is a crucial executor of apoptosis, cleaving and inactivating important cellular substrates such as (polyadenosine diphosphate-ribose) polymerase (PARP) [50]. Hence, to further understand the mechanism of apoptosis, activation of caspase-3 and PARP were evaluated.

### 2.7. Caspase-3/7 Quenching Assay

**Figure 7:**
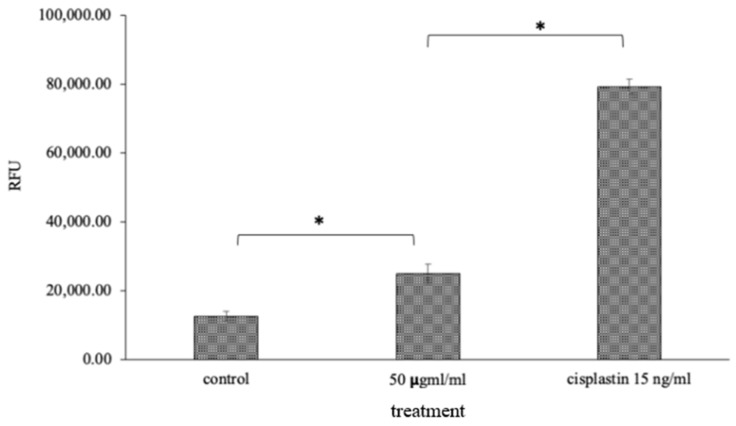
Caspase-3/7 quenching assay: 50 μg/mL of OI extract and Cisplatin, the standard drug, showed RFU value of 25,000 and 80,000, respectively, which may be due to an increase in the level of caspase 3 and 7 apoptotic proteins, which was significantly high compared to 10,000 RFU in the control (Figure 7). * *p* < 0.05, OI treatment v/s control and CP v/s OI.

The caspase family members participated in triggering different types of cell death. The amount of fluorescence is directly proportional to caspase-3/7 activity as indicated in Figure 7. Apoptosis is the course of programmed cellular death that manifests disassembling of the intracellular components while avoiding harm and inflammation of surrounding cells [51]. Caspases are involved in the regulation of inflammatory responses and cell death [52]. Caspase-3/7 is an effector caspase engaged in the death of dying cells’ final execution, whereas caspase-9 is an initiator caspase implicated in the intrinsic pathway [53,54].

### 2.8. Mitochondrial Stress Assay (Mitox Assay)

**Figure 8:**
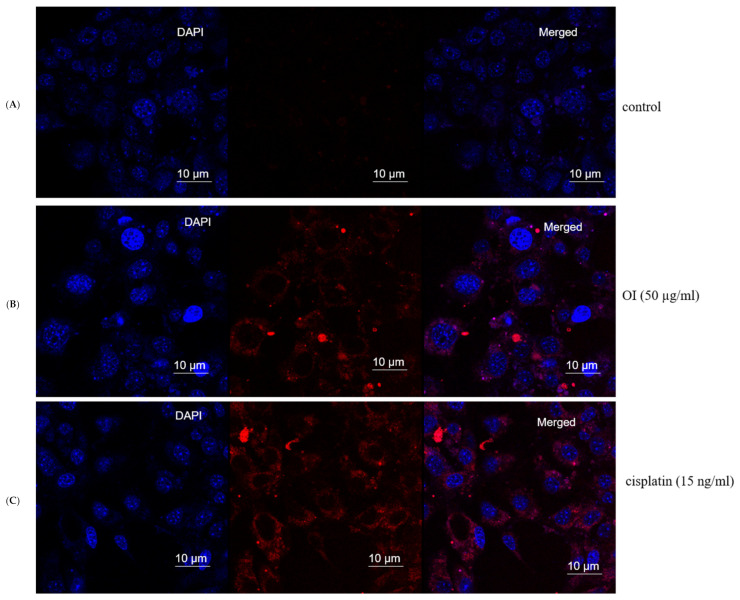
Mitochondrial stress assay (Mitox assay)—At 8 h, the nuclei of HSC-3 cells stained with DAPI fluorescent dye changed morphologically. (**A**) Control group. (**B**) (50 μg/mL) *Oroxylum indicum* treated HSC-3 cells stained more in blue colour indicating compact nucleus, nuclear blebbing and chromatin condenation (**C**) Cisplatin (15 ng/mL) was used as a positive control.

Nuclear damage and chromatin condensation, which are standard apoptotic markers, have been evaluated in HSC-3 cell lines treated with OI extract. Accumulated results indicate the induction of apoptosis by *O. indicum*. DAPI stains dsDNA by binding to A-T-rich regions in the minor groove of the DNA [55]. More DAPI penetrates the cell as the apoptotic cell membrane is damaged, staining the cell a darker blue tint (Figure 8B). Apoptotic cells’ different nuclear morphology, such as chromosome condensation and fragmentation, aids in the visual identification of apoptotic cells stained with DAPI. The OI extract-treated cells had a compacted nucleus and a loss of cell structure, whereas the untreated control cells looked to be normal (Figure 8A). Apoptotic cells labelled with DAPI also showed apparent nuclear blebbing, which can help identify necrotic cells from non-necrotic cells. DAPI staining has confirmed the hypothesis of a cell death phenomenon. These findings showed that *O. indicum* extract can efficiently trigger apoptosis in HSC-3 cells by causing chromatin and DNA damage.

### 2.9. Singlet Oxygen Production by OI in Mitochondria

**Figure 9:**
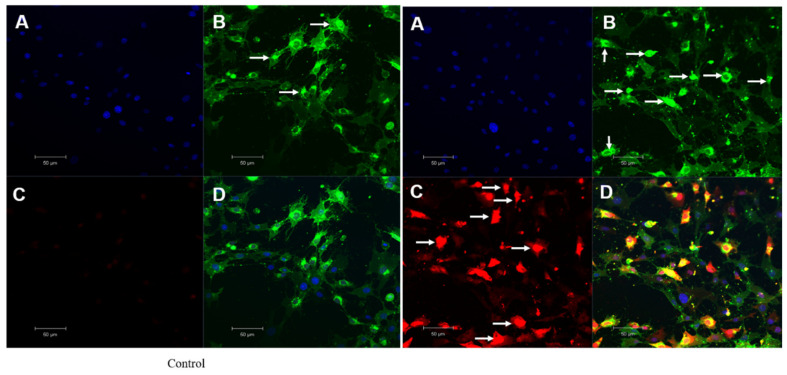
Mitochondrial singlet oxygen stress assay by triple-stain technique—comparing control and OI-treated HSC-3 cells. (**A**) nuclear stain by DAPI emits blue fluorescence. (**B**) viable cell by calcein green—the fluorescent calcein molecule is restored, which is trapped in the cell and emits strong green fluorescence. (**C**) mitostress by SiDMA stain—Si-DMA is able to selectively detect the 1O2 by emitting red fluorescence. (**D**) merger view shows both green and red fluorescence.

As Si-DMA is permeable to cells, a significant increase in fluorescence in mitochondria of OI-treated cells was observed, which indicates the increase in mitochondrial stress. Singlet oxygen production is always at mitochondria and is one of the routes in killing cancer cells. Si-DMA can selectively detect ^1^O_2_ (Figure 9). The measurement of singlet oxygen in living cells is critical for determining whether a medication has anticancer properties. In addition, using 5-aminolevulinic acid (5-ALA), a precursor of heme, Si-DMA can visualize the synthesis of ^1^O_2_ from protoporphyrin IX in mitochondria. Based on our results, we propose that OI extract works on singlet oxygen mitochondrial-mediated cell death. Control cells did not show mitochondria and Si-DMA-dependent fluorescent nature. Hence, we hypothesize that OI enters through the mitochondrial membrane and increases singlet oxygen production in HSC-3 cancer cells, thus causing DNA damage and finally leading to apoptosis.

### 2.10. Mitochondrial Membrane Potential (MMP) Bleaching Assay

**Figure 10:**
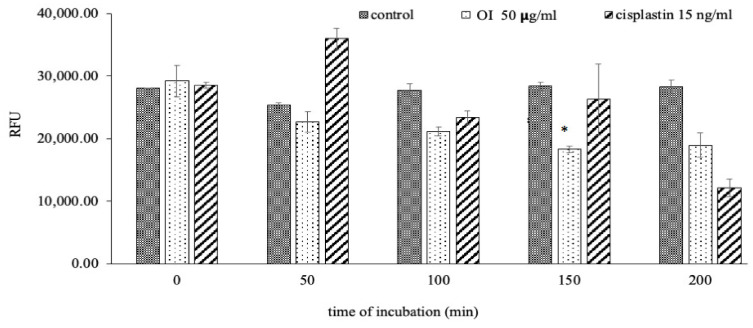
*O. indicum* extract alleviated mitochondrial membrane potential (MMP). Treatment of HSC-3 cells with OI extract (50 ug/mL) and cisplatin (15 ng/mL) at different incubation periods (0, 50, 100, 150 and 200 min). * *p* < 0.05 OI treatment v/s control at 150 min of incubation.

OI stem bark extract significantly reduced the level of RFU in HSC-3 cells at 150 min (Figure 10) of incubation compared to control. Our results are in agreement with the fact that a decrease in membrane potential is inversely proportional to apoptosis, and mitochondrial dysfunction has been shown to participate in the induction of apoptosis [56]. This result confirms that OI extract induces the disruption of mitochondrial membrane potential. Reduction in mitochondrial membrane potential by OI extract may well initiate the apoptotic cascade in the HSC-3, oral human-squamous carcinoma. It is well documented that ROS generation at a high level can lead to cellular damage by resulting in mitochondrial membrane damage, which can then induce toxicity [57,58]. One of the sequential phases in the apoptotic process is the disruption of the MMP. MMP level indicated that OI extract-induced cell death may be due to free radicals’ generation [59]. In a recent study, it was discovered that a similar event occurred [60]; treatment of *C. mutabilis* rhizome extract and Cm epoxide against human cancer cell lines resulted in phosphatidylserine externalization, increase in the levels of intracellular ROS, Ca^2+^, loss of mitochondrial membrane potential as well as fragmentation of genomic DNA.

In MMP assay, JC-1 dye penetrates and accumulates in the energetic and negatively charged mitochondria of healthy cells with a normal ΔΨM, forming red fluorescent J-aggregates spontaneously [61]. In apoptotic cells, however, the JC-1 dye reaches the mitochondria to a reduced extent, because the interior of the mitochondria is less negative due to greater membrane permeability and loss of electrochemical potential [62]. Based on these assumptions, the red/green fluorescence ratio of the dye in the mitochondria can be regarded as a direct indicator of the state of mitochondrial polarization, with the higher the ΔΨM, the greater the dye’s redshift. The smaller the ΔΨM of the mitochondria, the lower the red-to-green ratio of the fluorescent marker, and vice versa. As a result, a decrease in the red-to-green fluorescence intensity ratio indicates mitochondrial depolarization. Depolarization of the mitochondrial membrane exposes the mitochondrial permeability transition pore, which can lead to the release of apoptosis initiation factors like cytochrome c and trigger apoptosis cascade [63,64,65]; a drop in MMP could be linked to apoptosis pathways.

### 2.11. Colony Formation Is a Characteristic Phenomenon by OI

**Figure 11:**
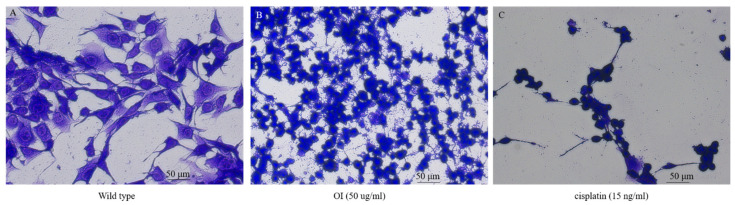
Colony formation assay in HSC-3 cell lines. (**A**) Control HSC-3 cells. (**B**) Reduction in the number of cell colonies on *O. indicum* (OI) treatment. (**C**) Cisplatin 15 ng/mL treatment shows a significant reduction in cell colonies.

The antiproliferative activity of *O. indicum* extract was evaluated using the clonogenic assay. This assay measures the potential of cells to expand into colonies unrestricted by growth contact inhibition—unlike normal growing cells that cease proliferation upon contact inhibition. As presented in Figure 11B,C, the colony formation declines with OI and standard drug, Cisplatin (15 ng/mL) treatment respectively.

### 2.12. OI Display Differential Phenomenon of Apoptosis Protein Expression

**Figure 12:**
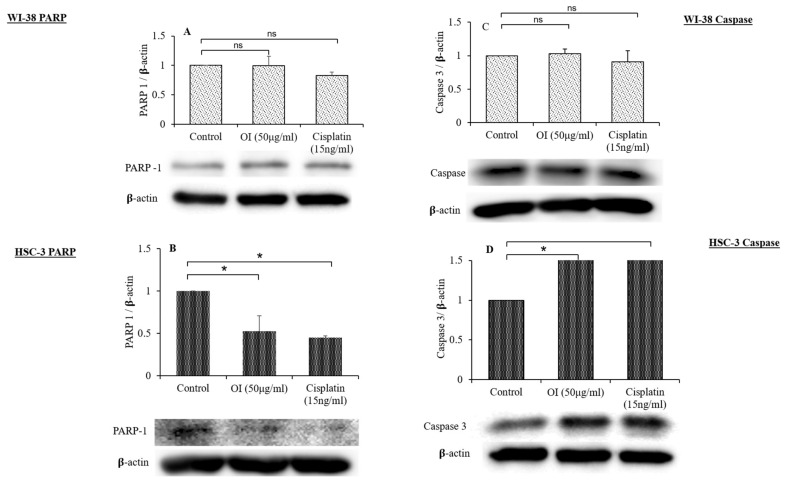
Western blot analysis of the effect of *O. indicum* extract on the expression of PARP and caspase-3. (**A**) PARP expression in WI-38 cell lines after 4 h exposure to OI extract. (**B**) Cleavage of PARP in HSC-3 cells after 4 h exposure to OI extract. (**C**) Expression of caspase-3 in WI-38 cell lines after 4 h exposure to OI extract. (**D**) Expression of caspase-3 in HSC-3 cell lines after 4 h exposure to OI. β-actin was used as a loading control. All Western blots are representative of three independent experiments. * *p* < 0.05, OI and CP v/s control; ns = non-significant changes, OI and CP v/s control.

To investigate the mechanism of apoptosis induction in HSC-3 cells and fibroblast cells by OI extract, Western blotting was used to evaluate the expression of anti-apoptotic (PARP) and pro-apoptotic (caspase-3) proteins (Figure 12).

The effect of OI extract treatment on HSC-3 cells showed the activation of caspase-3 (pro-apoptotic) and the cleavage of PARP (anti-apoptotic) relative to fibroblast cells, as shown in Figure 12A–D. As shown in Figure 12A,B, OI and CP could induce significant cleavage of PARP compared to control. The expression of caspase-3 in cells treated with OI and CP (Figure 12C,D) shows that both could induce a significant increase in the expression of caspase-3 compared to control. In our study, Western blot analysis revealed that progressive proteolytic cleavage products of PARP protein, a downstream target of activated caspase-3, occurred in HSC-3 cells treated with OI extract. PARP is a nuclear enzyme that is involved in the DNA repair process, and this 113 KDa protein is cleaved to 89 KDa and 24 KDa fragments by caspase-3 protease [66]. Treatment of HSC-3 cells with OI extract caused apoptotic cell death, as indicated by PARP cleavage.

Caspases are molecules that play an important part in the apoptosis process. Caspases are triggered in response to a signal and then act on their target molecules, resulting in cell death. Caspase-3 is a critical apoptosis protein that has been identified as a vital executioner caspase that is responsible for the activation and cleavage of over 100 substrates, ultimately leading to DNA fragmentation and apoptosis. Caspase-3 causes DNA fragmentation and death by cleaving the DNA repair enzyme poly-ADP-ribose polymerase (PARP) [67]. In our investigation, also caspase-3 and PARP, identified as important markers of apoptosis, indicated a considerable change in the expression on exposure to OI treatment. The results of Western blotting clearly showed PARP cleavage, which is a primary indicator of caspase-3 activation and PARP deactivation. Several prior investigations found that phytochemicals triggered apoptosis in malignant cells by activating caspase-3 and cleaving PARP [68].

DNA damage is caused by oxidative stress, which activates DNA repair enzymes. The activation of PARP-1 causes a decrease in cellular energy supply, which causes the cell to enter apoptosis by activating caspases, an apoptotic executor. Caspase-3 cleaves PARP-1 and disables its enzymatic activity, preventing cells from succumbing to energy deprivation and necrosis [69]. We used immunoblotting to investigate numerous cell death and stress indicators PARP-1, a DNA repair enzyme, is also activated when DNA damage is caused by oxidative stress. Mild oxidative stress causes DNA damage, which leads to PARP-1 activation, which causes excessive energy consumption and apoptosis. Excessive energy deprivation activates caspase-3 and cleaves PARP, preventing PARP overactivation. This causes the cells to enter apoptosis, which prevents necrosis from developing [70]. This was verified in our study by a considerable decrease in PARP-1 levels on exposure to OI extract.

## 3. Materials and Methods

### 3.1. Docking Studies

In silico computational studies were performed to understand the binding mode of the phytoconstituents with the target. Schrodinger 2020-4 suite device Maestro was used for in silico analysis. Docking of the phytoconstituents was carried out in the groove of the binding site of 5DS3 and 2CIE, which are the crystal structure of PARP protein and caspase enzyme, respectively. We have selected Oroxylin-A-7-*O*-beta-d-glucuronide ligand from the *Oroxylum Indicum* bark extract. The structures of the compounds were drawn in 2D sketcher [71]. Conversion of the chemical library from 2D to 3D structures, geometry optimization, selecting the bond orders and ionization state generation (through Epik) of the ligands were done by using the Ligprep module of Schrodinger suite-2020-4. OPLS-3E forcefield was used for the energy minimization of 3D structures of the ligands. The optimized ligands meeting all the requirements were further taken for docking studies [72].

### 3.2. Cell Culture Condition and Treatment

Human lung fibroblast (WI-38) and Human oral squamous cell HSC-3 (JCRB0623) cells were procured from the National Institute of Biomedical Innovation, Health and Nutrition (NIBIOHN, Osaka, Japan). Eagles modified basal medium (Nacalai Tesque, Tokyo, Japan)) with 10% FBS (Sigma-Aldrich, Saint Louis, MO, USA) and 5% antibacterial cock tail (Nacalai Tesque, Tokyo, Japan) was used to culture HSC-3 cell and M5S cells were cultured in α MEM with 10% FBS and 5% antibacterial cocktail. Cells were cultured in a sterile incubator with 95% air and 5% CO_2_ with saturated humidity at a temperature of 37 °C. All other chemicals used in this study were of analytical and molecular grade and purchased from Sigma-Aldrich (St. Louis, MO, USA). Clinical grade drug KEMOPLAT (Cisplatin injection, Dabur Pharma, New Delhi, India) was used as positive control drug.

### 3.3. End-Point Cell Toxicity Assay

Human oral squamosa cell HSC-3 (JCRB0623) cells were cultured in DMEM with FBS (10%) and 1% anti-bacterial cocktail (Nacalai, Tesque, Inc., Tokyo, Japan) up to sub-confluent stage in 96-well culture plates (Nunc, culture vessel, Thermo Fisher Scientific USA, Pittsburgh, PA, USA) at 37 °C, with a continuous supply of 5% CO_2_ and with maintenance of 95% humidity. Filter-sterilized (Sartorius Ltd., Göttingen, Germany) OI extract with different concentrations (0, 5, 20, 50, 100, 200, 500, 700 and 1000 ug/mL) were treated to HSC-3 cells at a fixed time of 12 h at standard culture condition. After incubation time, cells were washed twice with cold PBS, and MTT solution was added and incubated further for 4 h for the development of formazan crystals due to stimulation. After incubation time, DMSO solution was added to dissolve the formazan crystal and incubated at room temperature to develop a violet color. Finally, the intracellular purple-colored formazan formation was estimated using a spectrophotometer (λ_max_ = 570 nm), of make Multiskan FC, Thermo Fisher Scientific Inc., Pittsburgh, PA, USA). For time-depended *t*-cell proliferation assay, the same procedure was employed with a single dose of 50 ug/mL at different incubation periods of 0, 2, 4, 8 and 16 h. Percentage of cell viability was calculated with the following Formula (1).

(1) $$\mathrm{Cell}\;\mathrm{viability}\;\left(\%\right)\;=\;\mathrm{Abs}\;\mathrm{control}/\mathrm{Abs}\;\mathrm{Exp}\;\times \;100\;$$

### 3.4. Cell Morphometry Analysis

HSC-3 cells were treated with or without OI extract (50 ug/mL) and Cisplatin (15 ng/mL) for a 2 h incubation period in µ-Slide 4-well glass bottom chamber slides (Ibidi, GmbH, Lochhamer, Gräfelfing, Germany) at standard cell culture condition. Following treatment, cells were washed in PBS, fixed in ice-cold 100% methanol, and stained with 0.5% crystal violet (*w*/*v* 25% methanol) for 10 min. Cells were intensively washed with Milli Q water, mounted with glycerol and allowed to dry at room temperature. Photographs were taken using a bright-field microscope at 40× magnification (Olympus-1X-73, Olympus Corporation, Tokyo, Japan).

### 3.5. Calcein/EB Live-Cell Staining Assay

HSC-3 cells were treated with or without OI extract (50 ug/mL) and Cisplatin (15 ng/mL) for a 2 h incubation period in cover slip and cultured in standard cell culture condition up to 2 h. After the incubation period, 100 μL, 1% Calcein acetoxymethyl ester (Calcein AM, Anaspec, Inc., Fremont, CA, USA) and 50 μL of 0.5% ethidium bromide (Dojindo, Molecular technologies, Kumamoto, Japan) were added and incubated for 4 h. After the incubation period, cells were washed in cold PBS and fixed with 100% cold methanol. Fixed cells were further processed for fluorescent microscope experiments. The Florescent intensity was measured with Ex/Em of 490 nm/520 nm, respectively, for Calcein (green color) and Ex/Em 250 nm/605 nm for ethidium bromide (orange color). The green color indicates the live cells and the orange color indicates the dead cells. Cells were photographed at 20× by confocal microscopy (Leica, SP8 TCS, Solms, Germany).

### 3.6. DNA Ladder Assay

DNA break phenomenon was assessed by electrophoresis of extracted genomic DNA from HSC-3 cells treated with OI extract (50 ug/mL), as described previously with some modifications. Briefly, HSC-3 cells cultured in 6-well plates (1 × 10^6^ cells/mL) were treated with or without OI extract (50 ug/mL) for 4 h. After treatment, cells were washed with cold PBS and lysed with 250 μL of lysis buffer (10 mM EDTA, 50 mM Tris-Hcl, 0.5% SDS) in a hot water bath (40 °C). After digestion, cells were digested with proteinase-K (300 μg/mL), followed by the incubation with 200 μg/mL DNase-free RNase for 1 h DNA was extracted with 250 μL phenol: chloroform: isoamyl alcohol (25:24:1) for 1 min and centrifuged at 12,000 rpm for 10 min. The DNA in aqueous phase was further extracted with chloroform: isoamyl alcohol (24:1) and centrifuged at 12,000 rpm for 10 min. Soluble DNA in the aqueous phase was precipitated by adding 0.1 volume of 2 M NaCl and 2.5 volumes of chilled ethanol and kept at −20 °C overnight. The precipitated DNA was centrifuged at 12,000 rpm for 10 min and dissolved in cold Tris-EDTA buffer (pH 8.0) and electrophoresed in 1.0% agarose gel at 50 V for 1 h. The gel was photographed using LAS 4000 (Fujifilm, Tokyo, Japan).

### 3.7. Apoptosis Analysis by FACS

Annexin V-FITC apoptosis detection kit (Nacalai Tesque Inc., Kyoto, Japan) was used as per the manufacturer’s protocol, to detect apoptotic cell populations. Briefly, cells cultured in 6-well dishes were treated with OI extract (50 ug/mL) and Cisplatin (15 ng/mL) for a 2 h incubation period in 6-well culture plates. After the incubation period, cells were washed with cold PBS and collected by trypsinization using 0.5% trypsin-EDTA. Cells were centrifuged at 1000 rpm and single-cell suspensions were obtained by passing the cells through a 70 µm cell strainer (Bio Medical Science, Tokyo, Japan). Single-cell suspensions were incubated with Annexin V-FITC conjugate for 1 h at 4 °C, followed by Propidium Iodide for 15 min at room temperature. The percentage of live cells, early apoptotic, late apoptotic, and necrotic cells were analyzed by BD FACS caliber flow cytometer (BD Biosciences, Franklin Lakes, NJ, USA). Data were analyzed using BD Cell Quest^TM^ Pro (BD Biosciences).

### 3.8. Mitochondrial Singlet Oxygen Stress Assay

HSC-3 cells were cultured in standard cell culture condition with DMEM medium with 10% FBS, and treated with or without OI extract (50 ug/mL) for a 2 h incubation period in coverslip. After the incubation period, the medium was discarded and cells were washed twice with Hanks HEPES buffer and further treated with 100 μL of 50 nmol/L Si-DMA and 1% acetoxymethyl ester for 45 min at 37 °C. The nucleus was stained with DAPI. Cells were fixed and observed under the fluorescent microscope for green (Ex/Em of 490 nm/520 nm), red (Ex 650 nm/Em 685 nm) and blue (Ex 358/Ex 460 nm) images. Cells were photographed at 20× by confocal microscopy (Leica, SP8 TCS, Germany). All fluorescent images were merged intensity of yellowish orange, which indicates the mito-stressed cells due to OI treatment.

### 3.9. Mitochondrial Membrane Potential Assay

The potential of apoptosis due to mito stress was analyzed by JC-1 Mitochondrial membrane potential assay kit (JC-1 MitoMP, Dojindo Inc., Kumamoto, Japan). HSC-3 cells were cultured in standard cell culture condition with DMEM medium with 10% FBS and treated with or without OI extract (50 ug/mL) and cisplatin (5 ng/mL) for different time incubation periods of 0, 50, 100, 150 and 200 min in a 96-well culture vessel. After the incubation period, the medium was discarded and cells were washed twice with Hanks HEPES buffer, and cells were lysed with RIPA buffer and relative fluorescent unit (RFU) was noted with Ex 525 nm/Em 545 nm using a multimodal spectrofluorometer (Multiskan FC, Thermo Fisher Scientific Inc., Pittsburgh, PA, USA). Values were expressed in RFU vs. time of test compound incubation.

### 3.10. Caspase Quenching Assay

Time-resolved fluorescent quenching (true-point) caspase assay was done using the high-sensitivity DELFIA method (10). HSC-3 cells (1 × 10^4^ cells/mL) were cultured in 6-well culture plates with standard culture condition. Cells were incubated with OI (50 μg/mL) for a 4 h period. Untreated cells were designated as control cells. Another set of cells was treated with 50 ng/mL cisplatin, which served as positive control Europrium as a substrate for caspase-1 and samarium for caspase-3 were used as fluorescent labels. After the incubation period, fluorescent quenching analysis in terms of relative fluorescent unit (RFU) was carried out at emission of 642 nm and excitation of 312 nm.

### 3.11. Cell Transformation Assay

HSC-3 cells were cultured at a density of 1 × 10^3^cell/cm^2^ in a culture-suitable cover slip up to the semi-confluent stage. Cell transformation assay was conducted according to the method of Urani et al. [73]. Cells are treated with or without *Oroxylum indicum* (50 μg/mL) [C]. Positive control (15 ng/mL) for 7 days was carried out in standard culture conditions. Following treatment, cells were washed in PBS, fixed in ice-cold 100% methanol, and stained with 0.5% crystal violet (*w*/*v* 25% methanol) for 10 min. Cells were intensively washed with Milli Q water, mounted with glycerol and allowed to dry at room temperature. Photographs were taken using bright-field microscope at 40× magnification (Olympus-1X-73, Olympus Corporation, Tokyo, Japan).

### 3.12. Immunoblot Assay for Pro- and Anti-Apoptotic Proteins

Normal fibroblast (WI-38) was cultured in MEM with 10% FBS, and HSC-3 cells were cultured in DMEM (high glucose) with 10% FBS. After semi-confluent stage, cells were treated with or without test samples *Oroxylum indicum* (50 μg/mL) [C]. Positive control (15 ng/mL) for 4 h and total proteins were isolated by using selective extraction buffer (Protein Extraction Kit, Thermo Fisher Scientific, Waltham, MA, USA) as per the manufacturer’s protocol. Total protein concentrations were measured by the BCA method. Protein fractions were suspended in SDS-PAGE buffer containing 2-mercaptoethanol and boiled at 95 °C for 5 min. The protein samples were loaded onto 10% SDS-PAGE and subsequently transferred to PVDF membranes (GE Healthcare, Piscataway Township, NJ, USA). The membranes were blocked blocking solution in TBST (TBS and 0.1% Tween 20) for 1 h, and incubated overnight at 4 °C with specific primary monoclonal antibodies anti-PARP and Anti-Caspase-3 (Cell signaling Co, Danvers, MA, USA). The membranes were washed in TBST and further incubated with specific secondary antibodies for 1 h at room temperature. The protein bands were detected using an enhanced ECL kit (GE Healthcare, Tokyo, Japan) with the digital imaging system (LAS 4000, Fujifilm, Tokyo, Japan). Bands were measured using Image J software (NIH, Bethesda, MD, USA). Data are representative of three independent experiments; all experiments were performed in triplicate.

### 3.13. Statistical Analysis

All experiments were performed in triplicate with three independent experiments. Data are expressed as mean ± standard deviation. Mean differences between the groups were analysed by two-way ANOVA with post hoc Dunnett’s test with significant values of *p* < 0.05% against control. Significant differences were represented by single star.

## 4. Conclusions

In silico investigations employing the phytoconstituent, Oroxylin-A-7-*O*-beta-d-glucuronide identified in the *Oroxylum indicum* stem bark extract, docked effectively against PARP and caspase-3 proteins, implying that apoptosis in HSC-3 cancer cells might be induced. *Oroxylum indicum* stem bark extract increased cytotoxicity and subsequent cell death in a time-dependent manner. In HSC-3 cancer cells, this was accomplished via entry through the mitochondrial membrane, an increase in singlet oxygen production, phosphatidylserine externalization, an increase in intracellular ROS levels, loss of mitochondrial membrane potential, and fragmentation of genomic DNA, which collectively led to apoptosis. Nowadays, mitocons are gaining wide attention because they are well recognized anticancer drugs. Furthermore, because cancer stem cells have specific features that make them vulnerable to mitochondria-targeting medicines, *Oroxylum indicum* stem bark extract offers a viable option for therapeutic effect.

## Data Availability

This manuscript does not have any data-sharing criteria.

## Sample Availability

Samples of the compounds are available from the authors on request.
